# Characterization of patients that can continue conservative treatment for adenomyosis

**DOI:** 10.1186/s12905-021-01577-x

**Published:** 2021-12-28

**Authors:** Chiho Miyagawa, Kosuke Murakami, Takako Tobiume, Takafumi Nonogaki, Noriomi Matsumura

**Affiliations:** 1 Department of Obstetrics and Gynecology, Faculty of Medicine, Kindai University, 377-2 Ohnohigashi, Osaka-sayama, Osaka, 589-8511 Japan; 2 Department of Obstetrics and Gynecology, Osaka Red Cross Hospital, Osaka, Japan; 3 Department of Obstetrics and Gynecology, National Hospital Organization Osaka National Hospital, Osaka, Japan

**Keywords:** Adenomyosis, Hysterectomy, Treatment, Risk factors, Hormone

## Abstract

**Background:**

Historically, hysterectomy has been the radical treatment for adenomyosis. Although, some patients may not want to have their uterus removed, patients often have to no choice but to request hysterectomy during conservative treatment. The factors necessitating these hysterectomies remain unknown. The purpose of this study was to determine which patients can continue conservative treatment for adenomyosis.

**Methods:**

We selected women diagnosed with adenomyosis and provided with conservative treatment at the Kindai University Hospital and Osaka Red Cross Hospital in Osaka Japan from 2008 to 2017. Age at diagnosis, parity, uterine size, subtype of adenomyosis, type of conservative treatment, and timing of hysterectomy for cases with difficulty continuing conservative treatment were examined retrospectively.

**Results:**

A total of 885 patients were diagnosed with adenomyosis, and 124 started conservative treatment. Conservative treatment was continued in 96 patients (77.4%) and hysterectomy was required in 28 patients (22.6%). The cumulative hysterectomy rate was 32.4%, and all women had hysterectomy within 63 months. In the classification tree, 82% (23/28) of women aged 46 years or younger were able to continue conservative treatment when parity was zero or one. In those with parity two and over, 95% (20/21) of those aged 39 years and older had hysterectomy.

**Conclusions:**

Patients who continue conservative treatment for approximately 5 years are more likely to have successful preservation of the uterus. Multiparity and higher age at diagnosis are factors that contribute to hysterectomy after conservative treatment. Parity and age at diagnosis may be stratifying factors in future clinical trials of hormone therapy.

## Background

Adenomyosis is a benign disorder in which the endometrium and endometrial stromal cells proliferate in the muscle layer of the uterus [1, 2]. Associated symptoms of anemia, abdominal pain, and chronic pelvic pain due to excessive menstruation and dysmenorrhea are common in women of reproductive age and significantly impair quality of life [3]. Traditionally, adenomyosis was often first diagnosed by pathological examination after hysterectomy and was considered a disorder that affected the peri-menopausal period [4, 5]. However, with the widespread use of ultrasonography and magnetic resonance imaging (MRI) in recent years, it has become possible to accurately diagnose adenomyosis by imaging, and it is now diagnosed in relatively young women [6–9].

Historically, the radical treatment for adenomyosis has been hysterectomy [7]. However, conservative treatments of adenomyosis, such as hormone therapy and adenomyomectomy, are preferred by patients who are young and wish to preserve fertility, or do not want hysterectomy or may be at high risk for perioperative complications [10]. For women who would not like to become pregnant immediately, conservative treatment mainly involves hormone therapy, which is continued until menopause [11]. However, even with hormone therapy for adenomyosis, patients often experience persistent symptoms, including pain and drug side effects, such as irregular bleeding or osteoporosis, that result in the need for hysterectomy [10, 12–14]. To date, it has been unclear which women can continue conservative treatment for adenomyosis. The identification of factors related to the success or failure of conservative treatment would greatly contribute to the choice of treatment strategy, and significantly benefit the quality of life of women and the health care economy.

The purpose of this study was to evaluate the treatment course of patients with adenomyosis who have requested conservative treatment, and to determine which women can continue conservative treatment.

## Materials and methods

### Study design

Multi-institutional retrospective observation study.

### Cases

From January 2008 to December 2017, patients diagnosed with adenomyosis and started conservative treatment at Kindai University Hospital and Osaka Red Cross Hospital in Osaka Japan were selected and studied retrospectively. Exclusion criteria were the absence of symptoms due to adenomyosis (e.g., if the patient is being monitored for endometriosis or other comorbidities), absence of pre-treatment imaging, request for hysterectomy at the first visit, presence of submucosal myoma, presence of an intramyometrial myoma of more than 3 cm diameter, and/or presence of more than 3 fibroids that drain the endometrium were excluded because the later could cause heavy menstrual bleeding or abnormal uterine bleeding in addition to adenomyosis.

### Diagnosis

The diagnosis of adenomyosis was made using patients’ symptoms, such as dysmenorrhea and heavy menstrual bleeding, and imaging techniques, such as MRI or transvaginal ultrasound. The criteria for diagnosis by MRI were the presence of an enlarged myometrium with an indistinct limbus and a heterogeneous internal signal on T2-weighted images or thickening of the junctional zone (> 12 mm) [15, 16]. The diagnostic criteria for transvaginal ultrasonography were asymmetrical enlargement of the myometrium and an asymmetrical decrease in echogenicity of the lesion [1, 10]. Most of the cases were diagnosed by MRI, but only two cases were diagnosed by transvaginal ultrasonography without pre-treatment MRI. Age was defined as the age at the time when adenomyosis was diagnosed on imaging.

### Size measurement

**Fig. 1:**
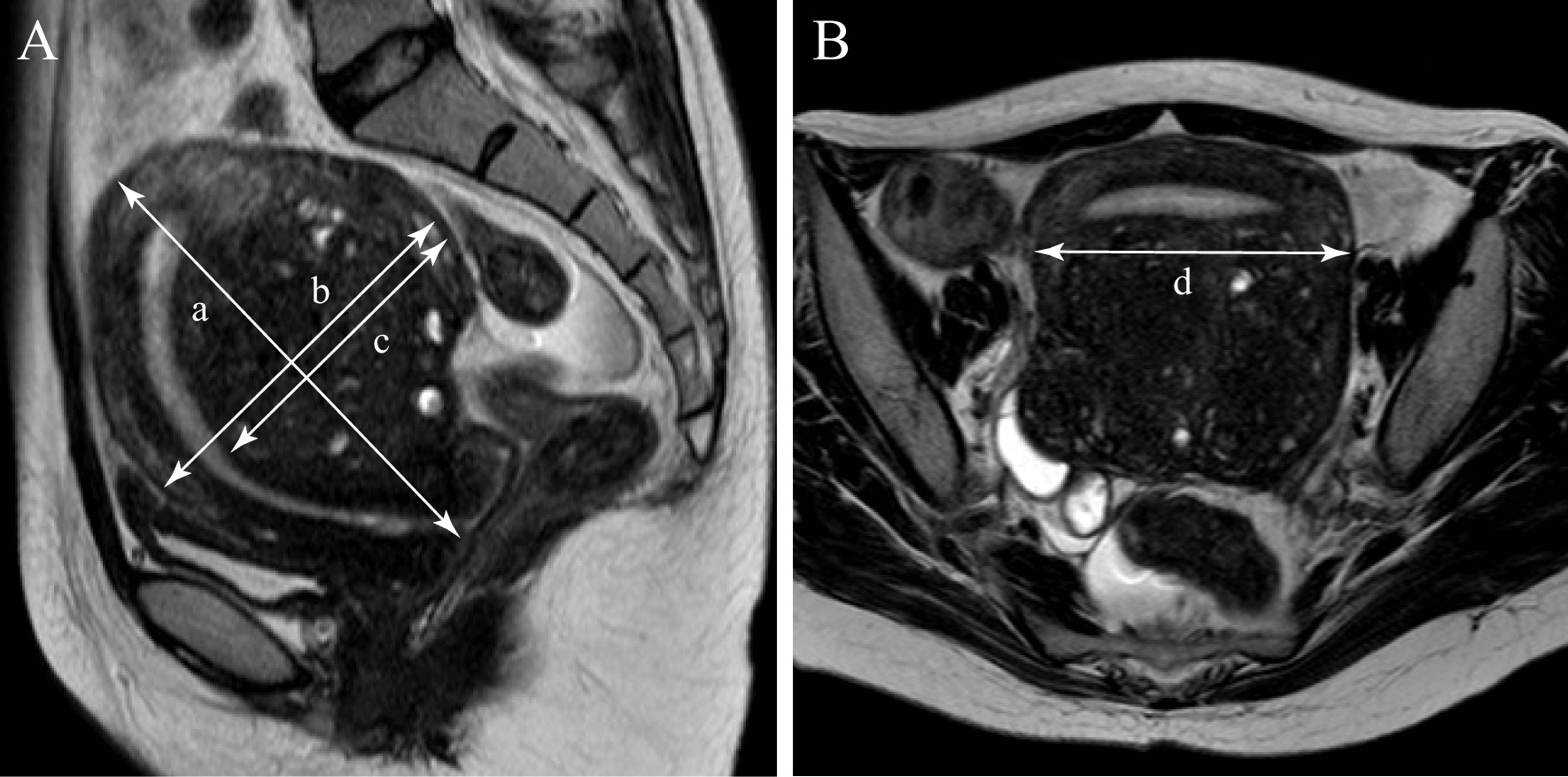
Measurement of uterine size. T2-Weighted Image (T2WI) of MRI. **A** We used the sagittal T2WI of the uterus to measure (a); the uterine long axis diameter, (b); the uterine short axis diameter and (c); the muscle layer thickness. **B** We used the axial T2WI of the uterus to measure (d); the uterine transverse diameter

Measurements of the size of the uterus and the myometrium were performed using MRI (Fig. 1). In sagittal sections of MRI T2-weighted images, the length from the cervix to the bottom of the uterus was defined as the long axis diameter of the uterus (a), the maximum diameter perpendicular to long axis diameter was defined as the short axis diameter of the uterus (b), and the thickness of the uterine muscle layer within the short axis diameter of the uterus was defined as the muscle layer thickness (c). The maximum transverse diameter of the uterus in the axial section of MRI T2-weighted images was defined as the transverse diameter of the uterus (d). In the two cases measured by transvaginal ultrasonography, (a), (b), and (c) were measured at the position of maximum sagittal section.

### Type of adenomyosis

Adenomyosis was classified into four subtypes based on MRI imaging features [17]. Subtype I adenomyosis involved adenomyotic lesions that extended from the endometrium and did not extend to the entire myometrium. Subtype II adenomyosis was defined as adenomyotic lesions that extended from the perimetrium and did not extend into the junctional zone. Subtype III adenomyosis was an isolated adenomyotic lesion in the myometrium that did not extend into the junctional zone and the perimetrium. Subtype IV adenomyosis was defined as a lesion that could not be classified as types I–III, where the lesion involved the entire muscle layer. Two cases diagnosed by transvaginal ultrasonography were not evaluated.

### Type of conservative treatment

Hormone therapy (gonadotropin releasing hormone agonist (GnRHa), progestins, levonorgestrel-releasing intrauterine system (LNG-IUS, Mirena intrauterine delivery system®, Bayer Yakuhin, Ltd), oral contraceptives (OCs), and danazol (BONZOL tablets®, Mitsubishi Tanabe Pharma Corporation) and adenomyomectomy were provided as conservative treatment for adenomyosis. Hysterectomy was performed after consultation with the patient when the symptoms worsened, or it became difficult to continue hormone therapy. Treatment was started on the date of the first visit, and the end of treatment was set at the date of the hysterectomy surgery or at the end of the observation period.

### Statistical analysis

Statistical analysis was performed using Graphpad Prism ver. 8.2.0 (GraphPad Software, San Diego, CA, USA). The cumulative hysterectomy rate was determined by the Log-rank test, and comparison between the two groups used the Mann–Whitney U test and χ^2^ test, with p < 0.05 as a significant difference. Classification tree was created using weka (https://doi.org/10.1016/j.knosys.2019.04.013).

## Results

**Fig. 2:**
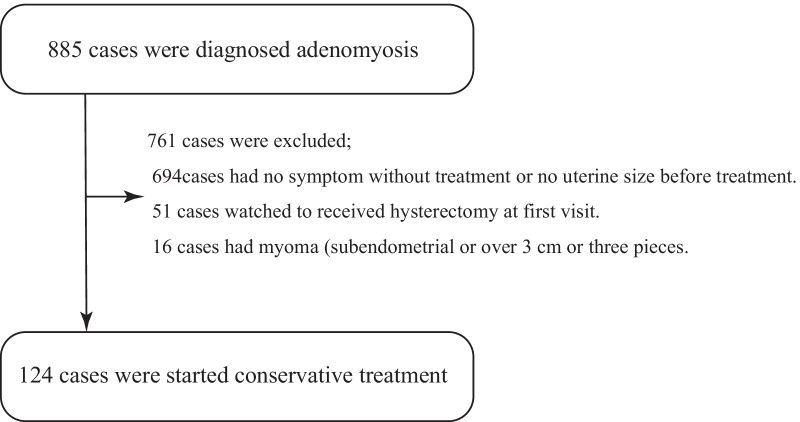
Cases flow chart. Of the 885 patients diagnosed with uterine adenomyosis, conservative treatment was initiated in 124 patients

**Fig. 3:**
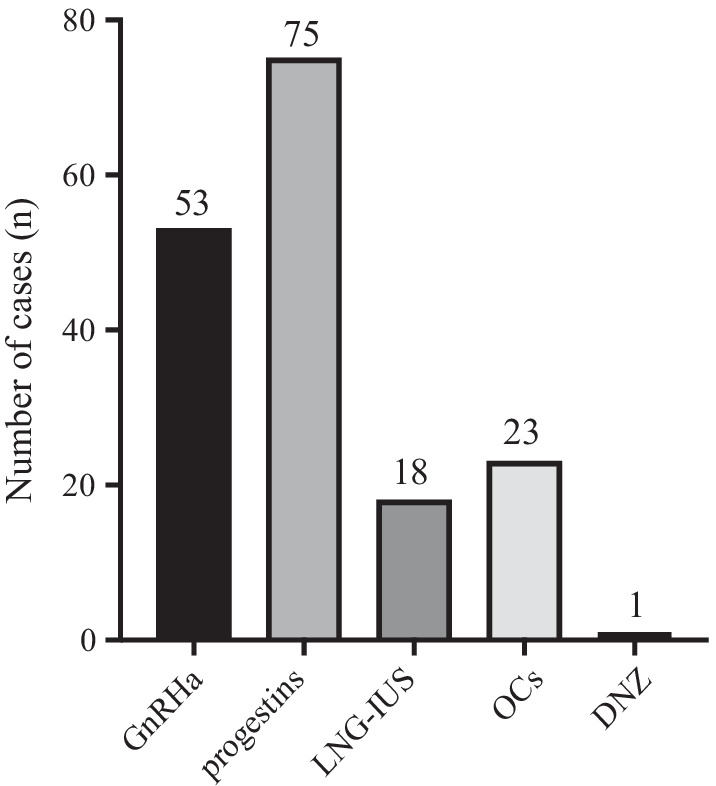
Number of cases treated with hormone therapy. GnRHa: gonadotropin releasing hormone agonist, LNG-IUS: levonorgestrel-releasing intrauterine systems, OCs: Oral contraceptives, DNZ: danazol. Y-axis shows the number of cases

A total of 885 patients were diagnosed with adenomyosis and started on treatment; 694 with no symptoms or no pre-treatment imaging, 51 who requested a hysterectomy at the time of first visit, and 16 with submucosal myoma or intramyometrial myoma of more than 3 cm and more than 3 fibroids, and 124 patients were started on conservative treatment (Fig. 2). Baseline characteristics of the 124 patients are presented in Table 1. The median treatment period was 28 months (1–132 months), median age was 41 years (24–53 years), median parity was 1 (0–3), median long axis diameter of the uterus was 9.7 cm (6.3–17.7 cm), median short axis diameter was 6.7 cm (3.5–12.9 cm), median transverse diameter was 6.8 cm (2.8–14.2 cm) and the median muscle layer thickness was 3.9 cm (1.3–8.8 cm). Adenomyosis subtypes I, II, III and IV were identified in 33 (26.6%), 28 (22.6%), 3 (2.4%) and 60 (48.4%) of these patients, respectively. Conservative treatment with hormone therapy alone was provided for 117 patients (94.4%), adenomyomectomy alone was performed for three patients (2.4%), and a mixture of these two procedures were provided for four patients (3.2%). The details of hormone therapy are presented in Fig. 3.

**Table 1:** Clinical characteristics of the 124 patients undergoing conservative treatmentfor adenomyosis

	n = 124
Age	41 (24–53)
Parity	1 (0–3)
Gravida	1 (0–6)
Size of uterus	
Long axis diameter (cm)	9.7 (6.3–17.7)
Short axis diameter (cm)	6.7 (3.5–12.9)
Transvers diameter (cm)	6.8 (2.8–14.2)
Muscle layer thickness (cm)	3.9 (1.3–8.8)
Type of adenomyosis	
Type I	33 (26.6%)
Type II	28 (22.6%)
Type III	3 (2.4%)
Type IV	60 (48.4%)
Type of treatment	
Hormonal therapy	117 (94.4%)
Adenomyomectomy	3 (2.4%)
Hormonal and adenomyomectomy	4 (3.2%)

**Fig. 4:**
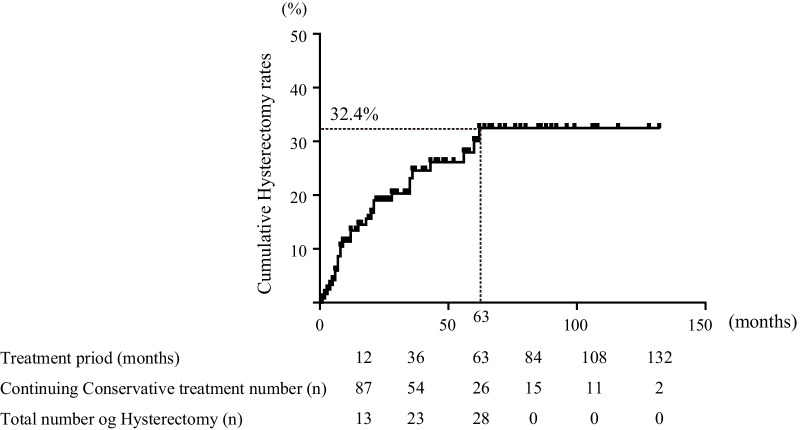
Cumulative hysterectomy rate. Kaplan–Meier analysis of the treatment period. The X-axis is the duration of treatment and Y-axis is the cumulative hysterectomy rate. The cumulative hysterectomy rate was 32.4% and reached a plateau after 63 months. The median treatment period was 28 months (1–132 months)

Ninety-six women (77.4%) were able to continue conservative treatment throughout the treatment period, and 28 patients (22.6%) required hysterectomy during conservative treatment. The cumulative hysterectomy rate, determined from the log-rank test of 124 patients who started conservative treatment, was 32.4% and the 28 that required hysterectomy (Group A) all had hysterectomy within 63 months (Fig. 4). Of the 96 patients who were able to continue conservative treatment, 26 were able to continue conservative treatment for adenomyosis beyond 63 months (Group B), and all of them ultimately did not require hysterectomy (Fig. 4).

The characteristics of Group A and Group B are presented in Table 2. Group A had a significantly higher age (Group A: 43 years, Group B: 37 years, p < 0.001), higher gravidity (Group A: 2, Group B: 0, p < 0.001) and parity (Group A: 2, Group B: 0, p < 0.001), and a significantly higher proportion of multipara (Group A: 82.1%, Group B: 42.3%, p < 0.001) compared with Group B. The long axis diameter (Group A: 11.1 cm, Group B: 9.0 cm, p < 0.001), short axis diameter (Group A: 7.7 cm, Group B: 6.0 cm, p = 0.002), transverse diameter Group A: 8.0 cm, Group B: 6.6 cm, p = 0.012), and muscle layer thickness (Group A: 4.6 cm, Group B: 3.5 cm, p = 0.018) were significantly larger in Group A than those in Group B. The proportion of patients with subtype IV adenomyosis and with the complication of endometriotic cysts were not significantly different between the two groups.

**Table 2:** Comparison of clinical characteristic of at baseline between cases failed conservative treatment (Group A) and continued uterine conservative treatment (Group B)

	Group A (n = 28)	Group B (n = 26)	p value
Age^†^	43 (33–53)	37 (27–46)	< 0.001
Gravida^†^	2 (0–6)	0 (0–3)	< 0.001
Parity^†^	2 (0–3)	0 (0–2)	< 0.001
Multipara^†^	23 (82.1%)	11 (42.3%)	< 0.001
Long axis diameter (cm)*	11.1 (7.6–17.7)	9.0 (6.4–13.0)	< 0.001
Short axis diameter (cm)*	7.7 (4.7–12.9)	6.0 (3.5–9.9)	0.002
Transvers diameter (cm)*	8.0 (4.1–14.2)	6.6 (3.7–9.2)	0.012
Muscle layer thickness (cm)*	4.6 (2.5–7.3)	3.5 (1.3–6.4)	0.018
Type IV adenomyosis*	18 (64.3%)	10 (38.5%)	0.059
Another of endometriosis*	11 (39.3%)	13 (50.0%)	0.766

*The Mann–Whitney U test, ^†^χ^2^ test

**Fig. 5:**
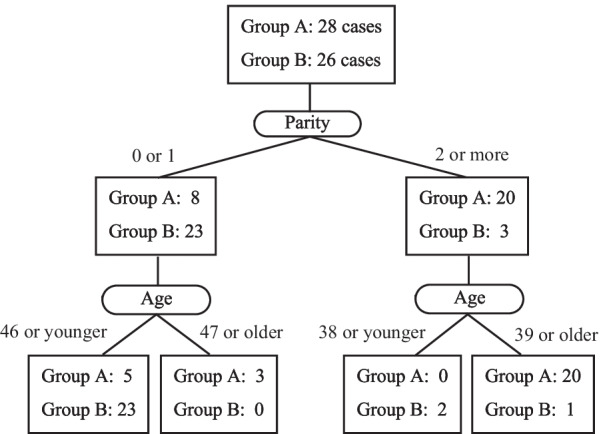
Classification tree. Group A; cases that required hysterectomy, Group B; cases that continued conservative treatment of adenomyosis. Accuracy: 77.8%

To determine the critical factors involved in whether conservative treatment for symptomatic adenomyosis can be continued or not, we produced a classification tree of Groups A (group of discontinued conservative treatment) and B (group of continued conservative treatment) using all the factors presented in Table 2, as shown in Fig. 5. Interestingly, only age and parity were factors that determined the need for hysterectomy. The first classified factor was parity, with 74% (23/31) of women with a parity of zero or one continuing conservative treatment, compared to only 13% (3/23) with a parity of two or more continuing conservative treatment. A total of 80% of patients were divided into two groups based on whether or not they could continue treatment with parity alone. For example, three cases of hysterectomy occurred in patients aged 47 years and older who had a parity of zero or one. When parity was two or more, only two patients younger than 38 years continued conservative treatment.

## Discussion

We retrospectively examined the course of attempted uterine preservation in patients with symptomatic adenomyosis to determine in which patient conservative treatment could be continued and in which patient hysterectomy was necessary. This study was unique in that (i) in patients who begin conservative treatment, the rate of hysterectomy increases until 5 years after initiation, after which it reaches a plateau, and (ii) the classification between uterine preservation and non-preservation was clarified by a classification tree. For the first time, this study showed that parity and age at diagnosis may be important factors for the consideration of conservative treatment for adenomyosis. Women in this study were relatively young, with a median age of 41 years, making them younger than those in reports from the early 2000s, but more consistent with recent reports [18–21]. The median parity was also low (at one), which may reflect the recent increase in aging of primipara and the trend of low fertility (United nations: World Population Prospects 2019). In previous reports examining the benefit of hormone therapy in adenomyosis, the mean pre-treatment uterine volume was 86 cm^3^ [22], 96.5 cm^3^ [3], 113.8 cm^3^ [23], 158.9 ml [24], 278 cm^3^ [25], and 311 cm^3^ [26]. The median uterine volume calculated from the long, short, and transverse uterine diameters in the present study was 217 (71–1400) cm^3^, so the size of the uterus was consistent with those previously reported. Adenomyosis was classified as subtype IV in half of the cases, which tended to be more severe than previously reported [17]. This may be due to the fact that the two centers participating in the study were core hospitals in the region, therefore accepting patients with advanced or difficult diagnosis. In conservative treatment for adenomyosis, adenomyomectomy is indicated when hormone therapy is difficult to continue or when the patient is undergoing infertility treatment. Because the uterine myometrium must be repaired after removal of the adenomyotic lesion, it is limited to lesions that are localized and capable of preserving the normal muscle layer [27]. In the present study, adenomyomectomy was chosen for a very small number of cases during infertility treatment or when there was a desire for surgery. Multiple methods of hormone therapy were used in most cases, including GnRHa, OCs, progestins, LNG-IUS and danazol. Multiple reports have shown that the smaller the size of the uterus at the start of hormone therapy, the more successful hormone therapy has been in treating adenomyosis [3, 22, 24, 25, 28, 29]. In this study, the size of the uterus at the start of treatment was also significantly smaller in Group B, which was able to continue with conservative treatment of adenomyosis (Table 1). However, previous reports have had mixed follow-up periods and may have included women who ultimately needed hysterectomy. In the present study, of the 124 patients who started conservative treatment, the failure to continue treatment and necessary hysterectomy were most frequent within the first year. This frequency then decreased, and treatment continued in all patients without much change until the fifth year. Women who were able to continue conservative treatment beyond 63 months did not require a hysterectomy. This novel analysis and the above results may provide guidance for planning the treatment of adenomyosis. Furthermore, our study exploring factors involved in the acceptability of conservative treatment found that patients undergoing conservative treatment for at least 5 years should be compared with those who have had hysterectomy. The success of conservative treatment requires a combination of long-term medical therapy or surgical therapy. In addition to the treatments used in this study, new GnRH antagonists are available [30–32], but as with GnRH agonists, the problem of side effects such as loss of bone density due to long-term administration remains. There are also some reports that uterine artery embolization and high-intensity focused ultrasound therapy are useful and safe as alternative therapies [33], but there is no randomized controlled trial, and the prognosis of pregnancy is unclear, so further verification is needed.

The classification tree was able to extract the fewest factors needed to separate the two patient groups (Groups A and B) most clearly in terms of sensitivity and specificity. Surprisingly, our current study revealed that uterine size and adenomyosis subtype classification [17] were not among the factors. Parity was one of the factors that classified the need to have hysterectomy or not, and most patients with a parity of two or more were found to eventually require hysterectomy. This may reflect the psychological factor of patients with two or more children wanting to prioritize parenthood, rather than continuing conservative treatment, which is also associated with symptoms such as irregular bleeding and pain. It has been reported that patients who had undergone hysterectomy for any condition, not just adenomyosis, were significantly more likely to have had a parity of two or more [4]. In addition, parity was reported to correlate with the incidence of adenomyosis [11, 33], which may have influenced this result. The another most factor in the classification tree was age at diagnosis. Parity and age at diagnosis may be related to the intensity of the desire to preserve the uterus, as well as the frequency of adenomyosis. It is known that the symptoms of adenomyosis in young patients are mainly dysmenorrhea, but heavy menstrual bleeding becomes the major symptom with age [34, 35]. However, it is difficult to interpret these in a univocal way by including them in a classification tree. It is helpful in daily practice to classify the possibility conservative therapy by objective indicators.

In future evaluations of pharmacotherapy for adenomyosis, age at diagnosis and parity may be important determinants of treatment success.

One limitation of this study was the small number of cases. We screened 885 cases of adenomyosis, but only 124 patients matched the criteria for inclusion in the analysis. In clinical practice, hormone therapy is often started based on clinical symptoms before MRI examination is performed, or diagnosis is made by ultrasonography alone. Therefore, in this study, it is likely that many cases did not undergo MRI before starting treatment for the same reason. Although the diagnosis of adenomyosis by ultrasonography is becoming more established [34, 35, 37, 38], it has been characterized by a lack of reproducibility and objectivity. In this retrospective study, there were very few cases in which the size was accurately measured by ultrasonography under reproducible conditions, so we excluded them from the study. The number of cases was further reduced to 26, because we found that only patients successfully treated for more than 63 months could be considered to have successful uterine preservation. Therefore, it is expected that about 5000 patients with adenomyosis would be needed to perform a similar analysis with more than 100 cases per group. Furthermore, this study was a retrospective study of routine practice over a 10-year period, and the diversity in treatments available over this period is also a limitation. In Japan, progestins and the levonorgestrel-releasing intrauterine system were approved within the last 5 years for the treatment of adenomyosis, the increased frequency of their use may have influenced the results. More than half of the patients had multiple cycles of treatment, making it difficult to determine the cause of discontinuation of conservative treatment. Although the diagnosis of adenomyosis by ultrasonography and MRI is being established [36–39], there are still many women who do not have a definitive diagnosis and are not treated even if they have symptoms. In addition, there are many early-stage cases is difficult to diagnose by ultrasonography or MRI. Therefore, to intervene appropriately, it is necessary to keep adenomyosis in mind.

## Conclusions

Uterine preservation in patients with adenomyosis is more likely to be successful if they can continue conservative treatment for approximately 5 years. In addition, multiparity and higher age at diagnosis are factors for hysterectomy during conservative treatment of adenomyosis. The results of this study may be useful in decision-making and for informed consent when treating patients with adenomyosis. Parity and age at diagnosis may be stratifying factor in future clinical trials on hormone therapy.

## Data Availability

The datasets generated and analyzed during the current study will be available from the corresponding author upon reasonable request.

## Abbreviations

MRI: Magnetic resonance imaging
GnRHa: Gonadotropin releasing hormone agonist
LNG-IUS: Levonorgestrel-releasing intrauterine system
OCs: Oral contraceptives
